# Immune-related gene data-based molecular subtyping related to the prognosis of breast cancer patients

**DOI:** 10.1007/s12282-020-01191-z

**Published:** 2020-11-27

**Authors:** Guoyu Mu, Hong Ji, Hui He, Hongjiang Wang

**Affiliations:** 1 Breast Surgery Department, The First Affiliated Hospital of Dalian Medical University, Dalian, 116000 Liaoning China; 2 Gynecology and Obstetrics Department, The Second Affiliated Hospital of Dalian Medical University, Zhongshan Road 467, Dalian, 116023 Liaoning China; 3 Department of Laparoscopic Surgery, The First Affiliated Hospital of Dalian Medical University, Dalian, 116000 Liaoning China

**Keywords:** Breast cancer, Immunotherapy, TCGA database

## Abstract

**Background:**

Breast cancer (BC), which is the most common malignant tumor in females, is associated with increasing morbidity and mortality. Effective treatments include surgery, chemotherapy, radiotherapy, endocrinotherapy and molecular-targeted therapy. With the development of molecular biology, immunology and pharmacogenomics, an increasing amount of evidence has shown that the infiltration of immune cells into the tumor microenvironment, coupled with the immune phenotype of tumor cells, will significantly affect tumor development and malignancy. Consequently, immunotherapy has become a promising treatment for BC prevention and as a modality that can influence patient prognosis.

**Methods:**

In this study, samples collected from The Cancer Genome Atlas (TCGA) and ImmPort databases were analyzed to investigate specific immune-related genes that affect the prognosis of BC patients. In all, 64 immune-related genes related to prognosis were screened, and the 17 most representative genes were finally selected to establish the prognostic prediction model of BC (the RiskScore model) using the Lasso and StepAIC methods. By establishing a training set and a test set, the efficiency, accuracy and stability of the model in predicting and classifying the prognosis of patients were evaluated. Finally, the 17 immune-related genes were functionally annotated, and GO and KEGG signal pathway enrichment analyses were performed.

**Results:**

We found that these 17 genes were enriched in numerous BC- and immune microenvironment-related pathways. The relationship between the RiskScore and the clinical characteristics of the sample and signaling pathways was also analyzed.

**Conclusions:**

Our findings indicate that the prognostic prediction model based on the expression profiles of 17 immune-related genes has demonstrated high predictive accuracy and stability in identifying immune features, which can guide clinicians in the diagnosis and prognostic prediction of BC patients with different immunophenotypes.

**Electronic supplementary material:**

The online version of this article (10.1007/s12282-020-01191-z) contains supplementary material, which is available to authorized users.

## Background

Breast cancer (BC), which is the most common malignant tumor and the leading cause of cancer-related deaths in women in underdeveloped countries, affected 882,900 individuals and resulted in 324,300 deaths in 2012 alone; that year, BC accounted for 25% and 15% of all cancer cases and cancer deaths among females, respectively [1]. Generally, BC is associated with reproductive and endocrine risk factors, including oral contraceptive use, nulliparity and long menstrual periods [2]. On the contrary, some potentially modifiable risk factors include alcohol consumption, obesity, physical inactivity, and menopausal hormone therapy [3].

Some large-scale clinical data indicate that systemic adjuvant chemotherapy should generally not be recommended for most patients with early BC following surgery or radiotherapy, since chemotherapy would result in far greater toxicity relative to the survival benefit of the patients [4–6]. However, patients with a low likelihood of survival who do not undergo chemotherapy will quickly relapse, which results in the invasion of adjacent tissues and distant metastasis [7]. Consequently, it is particularly important to determine the relevant survival risk of patients through subgroup classification and early diagnosis and to provide additional systemic adjuvant chemotherapy to high-risk patients.

According to recent studies, BC can be classified into the following four subtypes: Luminal A (ER + /PR + /HER2 −, grade 1 or grade 2), Luminal B (ER + /PR + /HER2 + , or ER + /PR + /HER2 − grade 3), HER2-overexpressing (ER − /PR − /HER2 +), and triple-negative breast cancer (TNBC, ER − /PR − /HER2 −) [8]. Among them, the Luminal A subtype is associated with a favorable prognosis and sensitivity to endocrine therapy, which means that only endocrine therapy is the general treatment approach [9]. On the contrary, the Luminal B subtype is associated with a high tumor proliferation rate. The HER2-negative Luminal B subtype can usually be treated with endocrine therapy + chemotherapy, while the HER2-positive Luminal B subtype is generally treated with chemotherapy + anti-HER2 treatment + endocrine therapy [10]. Moreover, the HER2 overexpressing subtype is characterized by a poor prognosis and rapid progression and is mainly treated with chemotherapy + anti-HER2 therapy [11]. Specifically, the negative expression of ER, PR and HER2 in TNBC is related to its unique biological characteristics and potent heterogeneity, and the only standard treatment recommended for this subtype is chemotherapy [12]. Recently, progress has been made in the early diagnosis and treatment of BC, which makes BC a treatable disease; however, multidrug resistance (MDR) remains a major challenge in the treatment of metastatic BC, as the typical survival time of patients with metastatic BC is only 2–3 years [13]. Unfortunately, this general classification method cannot accurately reflect individual differences [14]. It is worth noting that the existing large-scale databases that contain gene expression data, including the TCGA and ImmPort, enable us to search for potentially reliable BC biomarkers to predict and classify patient prognosis [15].

Increasing evidence has supported the idea that immunocytes in the tumor microenvironment can remarkably promote or inhibit tumor growth, and thus, they can serve as indicators of BC prognosis. In addition, immune escape has been verified as a novel cancer marker [16]. In recent years, through immunotherapies, such as the BC vaccine, monoclonal antibodies (MAb), antibody–drug conjugates (ADCs), checkpoint inhibitors and stimulating molecule agonist antibodies, great progress has been achieved in the treatment of BC patients [17–20]. Moreover, tumor-infiltrating lymphocytes (TILs) and tumor-related macrophages in BC tissues have also been found to have crucial functions in the immune escape mechanism of tumor cells, and thus, they are remarkably related to patient prognosis [21, 22]. Nonetheless, the molecular events of tumor cell–immunocyte interaction in the BC microenvironment should be further examined and summarized, as the contribution of these events and their potential roles in predicting the prognosis of BC patients should be determined [23].

In this study, a prognostic prediction model for BC was developed and verified based on immune-related genes retrieved according to the clinical features of patients whose data were collected from the TCGA and ImmPort databases. Our findings are promising in that they may help clinicians evaluate the prognosis and therapeutic options for BC patients as well as therapeutic effects.

## Materials and methods

### Preprocessing of preliminary sample data and initial screening of BC immune-related genes

The most recent clinical follow-up data were downloaded on December 14, 2018 through the TCGA GDC API. In all, 1222 RNA-Seq data samples were included, as shown in Table S1. Overall, 1109 of these 1222 data samples were tumor tissues, while the remaining 113 were normal tissues. In addition, an immune-related gene set, which covered 1811 genes, was also downloaded from the ImmPort database on October 8th, 2018, as shown in Table S2.

First, the retrieved 1109 RNA-seq data samples were preprocessed according to the steps described below: (1) 39 samples with no clinical data and 21 with 0 OS (overall survival) were removed, (2) the normal tissue sample data was removed, (3) genes of FPKM (Fragments Per Kilobase Million) < 1 were also removed from all samples, and (4) only the expression profiles of immune-related genes were preserved. Altogether, 1376 genes were used for the subsequent analysis of the model. The preprocessed data are shown in Table S3, while the sample statistics of the clinical information are displayed in Table 1.

**Table 1:** Sample statistics of training set and test set

Clinical Features	Overall	Train	Testing	*p* value
OS	1068	533	535	0.862408
T	1068	533	532	0.356377
T1	279	155	124	
T2	616	291	325	
T3	132	70	62	
T4	38	17	21	
TX	3	0	3	
N	1068	526	525	0.613292
N0	502	256	246	
N1	357	182	175	
N2	119	58	61	
N3	73	30	43	
NX	17	7	10	
M	1068	461	444	0.688259
M0	883	451	432	
M1	22	10	12	
MX	163	72	91	
Stage	1068	521	525	0.424994
I	181	106	75	
II	606	297	309	
III	239	109	130	
IV	20	9	11	
X	22	12	10	
Age	1068	533	535	0.515704
0 ~ 40	75	42	33	
40 ~ 50	219	118	101	
50 ~ 60	283	143	140	
60 ~ 70	277	124	153	
70 ~ 100	214	106	108	
IHC_Her2	1068	306	296	0.701374
0	59	31	28	
1 +	263	134	129	
2 +	194	101	93	
3 +	86	40	46	

Second, 1068 samples were classified into the training set and test set, and random grouping with replacement was performed for all samples for 500 times in advance to eliminate the impact of random allocation bias on model stability. Grouping was performed based on the training set: test set ratio of 0.7:0.3 since the BC sample size was over 1000. Specifically, the most suitable training and test sets were selected based on the following criteria: (1) similar age distributions, clinical stages, follow-up times and death proportions between the two groups; and (2) close binary sample sizes in the two randomly divided datasets after clustering the gene expression profiles. The final training set data (*n* = 533) are displayed in Table S4, and the test set data are shown in Table S5 (*n* = 535). Moreover, the clinical information statistics of both the test set and training set samples are presented in Table 1. The final information of both the training set and test set samples is shown in Table 1. No significant difference was observed between the training set and test set data, as verified by the *P* value, which indicated reasonable sample grouping.

### Single-factor survival analysis of immune-related genes in the training set

All immune-related genes were analyzed using the univariate Cox proportional hazards regression model; at the same time, survival data were evaluated by the survival coxph function of R software [24], and *p* < 0.05 served as the significance threshold.

### Screening of specific immune-related genes for BC prognosis and construction of the prognostic prediction model

First, the least absolute shrinkage and selection operator (Lasso, Tibshirani, 1996) algorithm was used to further narrow the range of prognosis-specific immune-related genes under the condition of maintaining high accuracy. Moreover, the glmnet package of R software was used for the lasso Cox regression analysis. Next, to further compress the number of immune-related genes, the R package MASS was employed for stepwise regression analysis using the Akaike information criterion (AIC), which considered the degree of fit of the statistical model as well as the number of parameters used in fitting. The StepAIC method in the MASS package originated from the most complex model, in which one variable was deleted sequentially to reduce the AIC; a smaller value was indicative of a superior model, which demonstrated a sufficient degree of fit and fewer parameters of the model. The risk model of 17 genes (Table S7) was finally obtained using this algorithm. The results of the stepwise regression are presented in Table S8. The formula was as follows:

RiskScore = PIK3CA*0.025861691 + CCR7*0.014541227 + SEMA7A*0.158263093 + ACVR2A* − 0.437173332 + CBL*0.231921725 + PLXNB2*0.014940811 + PLXND1*0.033074364 + APOBEC3F* − 0.314321194 + NFATC2* − 0.257156537 + NFKBIZ* − 0.046977178 + TNFSF4*0.16976996 + DAXX* − 0.034395422 + TLR2*0.023037905 + SEMA3B* − 0.044973358 + HSPA2* − 0.023131493 + TPT1* − 0.001623522 + CCL22* − 0.077745415.

Afterwards, the expression profiles of related genes were collected from both the training set and test set; subsequently, they were incorporated into the model to calculate the RiskScore of all the samples. Then, the median RiskScore served as the threshold by which the samples were classified into either the high-risk group (Risk-H) or the low-risk group (Risk-L); afterwards, a receiver-operating characteristic (ROC) curve analysis, Kaplan–Meier (KM) analysis and gene-clustering analysis were performed to comprehensively assess the efficiency, accuracy and stability of the model in predicting and classifying the prognosis of BC patients.

### Functional annotations and signaling pathway enrichment of immune-related genes specific for prognosis

The gene families of the 17 screened genes were annotated according to the human gene classification in the HGNC (Human Gene Nomenclature) database [25]. Specifically, the clusterProfiler package of R software was used for the KEGG (Kyoto Encyclopedia of Genes and Genomes) and GO (Gene Ontology) enrichment analyses for the abovementioned 17 immune-related genes specific for prognosis. Specifically, the gene sets that intersected with the 17 genes were compared in each GO term and KEGG pathway. The GO term or KEGG pathway was considered annotated by the genes if there was an intersection, and finally, all the GO terms and KEGG pathways that annotated to the 17 genes were obtained.

### Correlation between the RiskScore and signaling pathways as well as the clinical features of the samples

First, the KEGG functional enrichment scores of all samples were analyzed using the single-sample gene set enrichment analysis (ssGSEA) function of the R software package GSVA [26]. In addition, the correlation with the RiskScore was calculated, and a clustering analysis was performed according to the enrichment score of each sample in each pathway.

Subsequently, the correlations of related factors (including T, N, M, Stage, Age and HER2 expression) with the RiskScore were evaluated. Then, the nomogram model and forest plot were established using the clinical features (such as T, N, M, Stage, Age and HER2 expression) as well as the RiskScore, and the correlations of the RiskScore and the various clinical features with patient survival were assessed. The analysis process is shown in the Figure workflow.
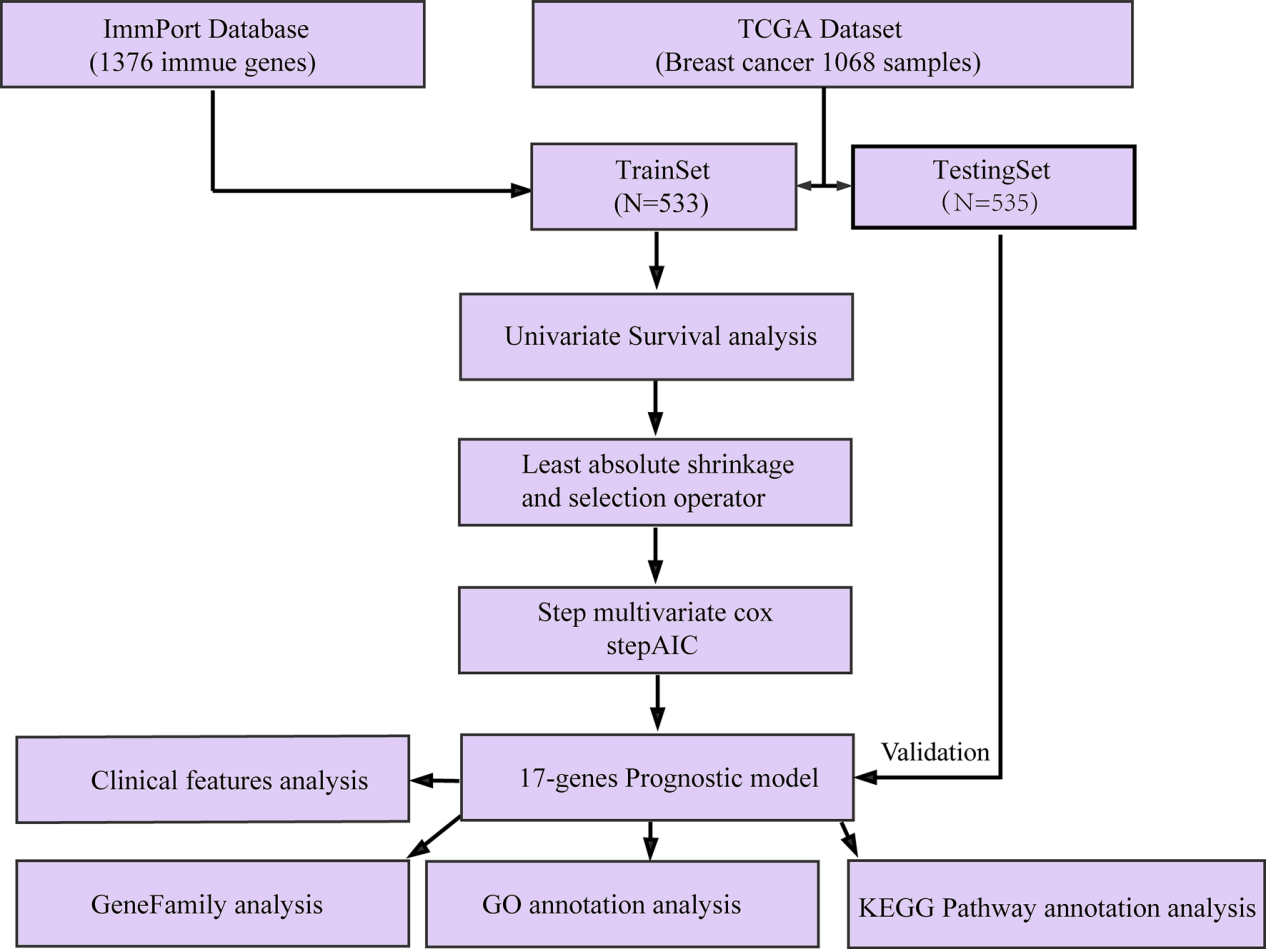


## Results

### Retrieval of immune-related genes based on the survival and prognosis of BC patients

**Fig. 1:**
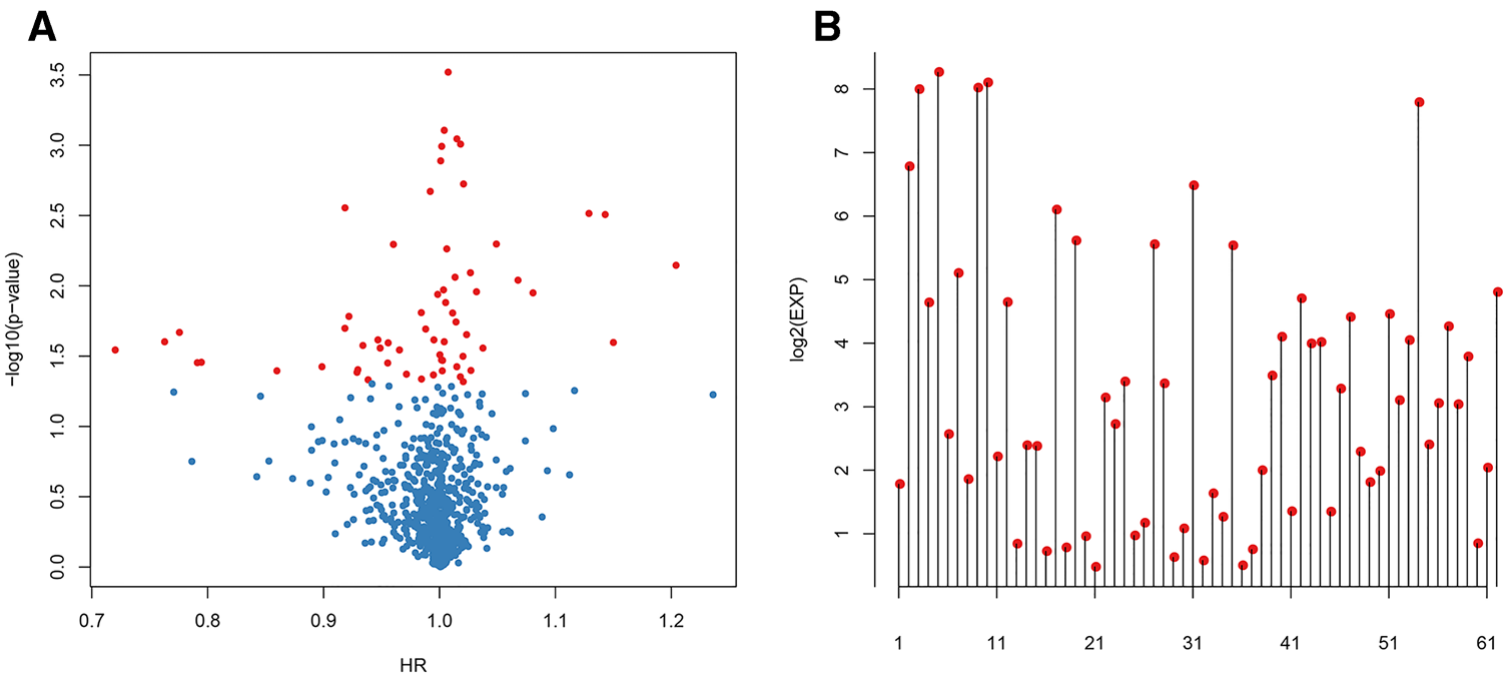
The relationships between the *p* values of 62 genes and the HR and expression levels. **a** The relationships of the *p* values of 62 genes and the HR is shown. **b** The relationships of the *p* values of 62 genes and the expression levels. Red dots represent significantly different immune-related genes (*p* < 0.05) associated with prognosis

First, related data were downloaded from the TCGA and ImmPort databases and were then preprocessed (see “Materials and methods”). Subsequently, all the immune-related genes and survival data were analyzed by a univariate Cox proportional hazards regression model using the survival coxph function of R, with the significance level set at *p* < 0.05, as shown in Table S6. Eventually, 62 significantly different immune-related genes that were also associated with prognosis were discovered. The relationships of the *p* values of these 62 genes with the HR and expression levels are shown in Fig. 1.

### Screening of prognosis-specific immune-related genes and construction of the prognostic prediction model for BC

Sixty-two immune-related genes were recognized, but many of these genes are not suitable for clinical detection. Consequently, the scope of immune-related genes was further narrowed to guarantee high accuracy. Thus, the R software package glmnet was used for the lasso Cox regression to refine the prognostic genes identified above, which led to a reduction in gene numbers from 62 to 29. Moreover, the R package MASS was employed for stepwise regression analysis using the AIC, which considered the degree of fit of the statistical model and the number of parameters used for fitting. On the contrary, the StepAIC method in the MASS package originated from the most complex model, in which one variable was deleted sequentially to reduce the AIC; a smaller value suggested a superior model, which indicates a sufficient degree of fit and fewer parameters of the model. Finally, the risk model of 17 genes was obtained using this algorithm (Table S7). The formula is provided in the “Materials and methods”.

**Fig. 2:**
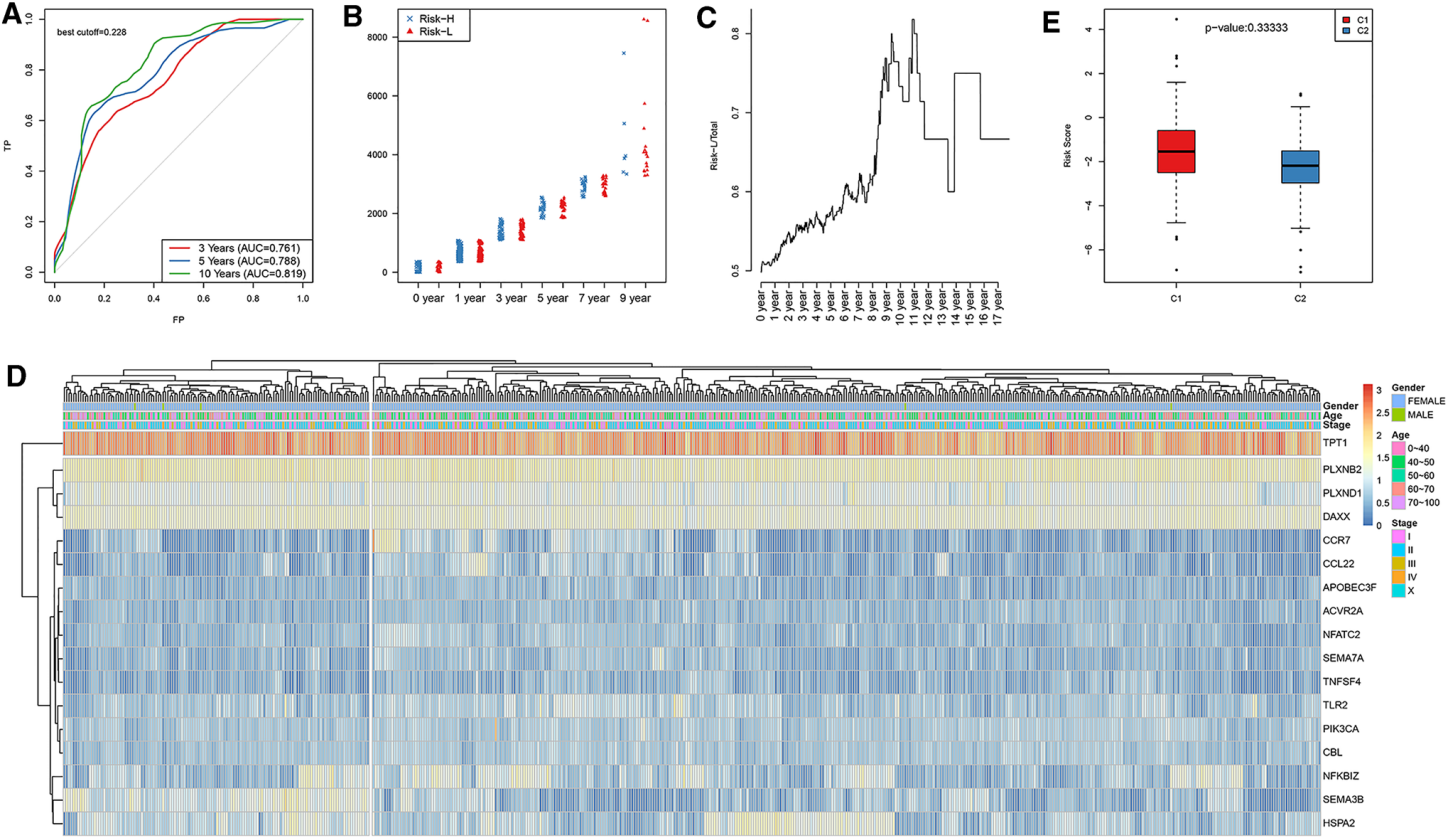
Verification of the stability of the prognostic prediction model included 17 immune-related genes for the BC patient training set. **a** The predicted survival according to the ROC curves of the 17-gene risk model in the training set. **b** The distribution of samples in the Risk-H and Risk-L groups of the training set divided through the 17-gene risk model under different OS periods. **c** The level of Risk-L group/total sample size with the extension in OS in the training set. **d** The clustering results of the training set samples. **e** Difference in the RiskScore between the two groups, which were clustered by the expression of 17 genes in the training set samples

Subsequently, training set samples were incorporated into the formula to calculate the RiskScore for all the samples, and the median RiskScore served as the threshold by which the samples were divided into either the high risk (Risk-H) or low risk (Risk-L) group. Furthermore, ROC curve analysis of the prognostic classification for the RiskScore was performed using the survivalROC package of R software. The OS distribution of the samples was approximately > 2 years (Fig. S1); as a result, the model predicting effect for the 3-, 5- and 10-year survival was evaluated in this study, with an average AUC of approximately 0.789, as presented in Fig. 2a. In addition, the sample distribution in the Risk-H and Risk-L groups under different OS periods is presented in Fig. 2b. As could be observed, no obvious difference in sample size was detected between the 0- and 1-year OS of the Risk-H and Risk-L groups; moreover, the sample size in the Risk-H group after the 5th year was dramatically smaller than that in Risk-L group, which had become markedly significant as the OS extended (Fig. 2c). The clustering results of the training set samples are presented in Fig. 2d. Obviously, the abovementioned 17 genes could be markedly clustered into high and low expression groups, while samples in the training set could also be assigned to two groups; the RiskScore values of the two subclasses were also compared (Fig. 2e).

**Fig. 3:**
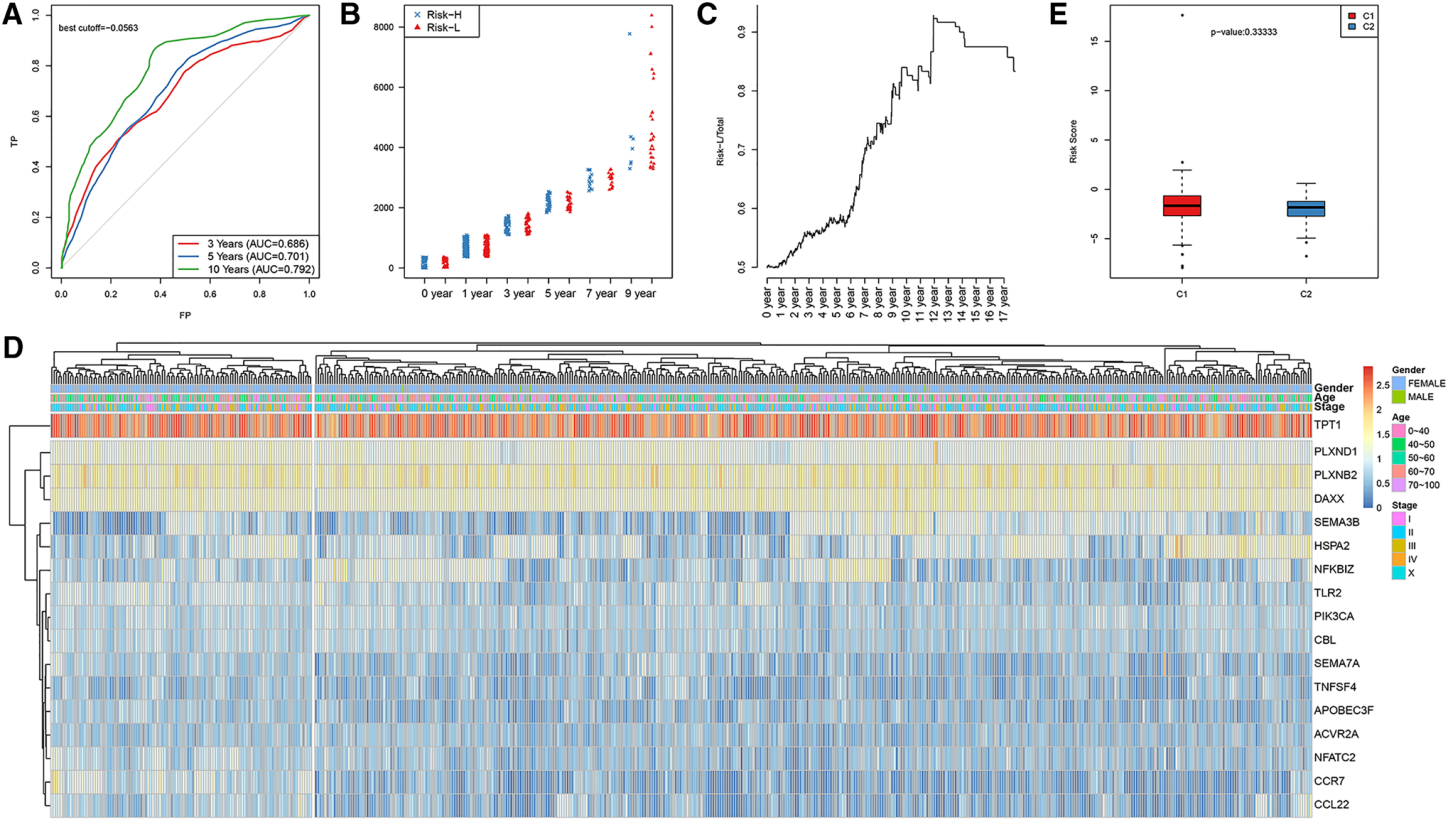
Verification of the reliability of the prognostic prediction model included 17 immune-related genes for the BC patient test set. **a** The survival predicted by the ROC curves of the 17-gene risk model in the test set. **b** The distribution of samples in the Risk-H and Risk-L groups of the test set divided through the 17-gene risk model under different OS periods. **c** The level of Risk-L group/total sample size with the extension in OS in the test set. **d** The clustering results of the test set samples. **e** Difference in the RiskScore between the two groups, which were clustered by the expression of 17 genes in the test set samples

In addition, to further confirm the stability and reliability of the prognostic prediction model, the expression profiles of these 17 genes were obtained from the test set and then integrated into the model for model verification; at the same time, the RiskScore of the samples was also calculated. Afterwards, data in the test set were used to evaluate the ability of the model to predict the 3-, 5- and 10-year survival rates. As shown in Fig. 3a, the average 3–10-year AUC is 0.726. The sample distribution in both the Risk-H and Risk-L groups at different OS periods is also displayed in Fig. 3b. No significant difference was observed in OS between the Risk-H group and Risk-L group at 0 and 1 year; in addition, the sample size in the Risk-H group after the 3rd year was notably reduced compared with that in the Risk-L group, which became more obvious as the OS increased (Fig. 3c). The clustering results for the samples in the test set and the difference in RiskScore values between the two groups are shown in Fig. 3d and e, respectively.

**Fig. 4:**
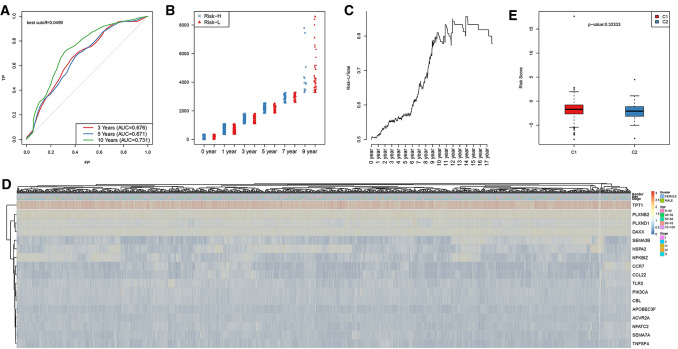
Verification of the reliability of the prognostic prediction model included 17 immune-related genes for all the BC patients in both sets. **a** The survival predicted by the ROC curves of the 17-gene risk model. **b** The distribution of all the samples in the Risk-H and Risk-L groups divided through the 17-gene risk model under different OS periods. **c** The level of Risk-L group/total sample size with the extension in OS. **d** The clustering results of all the samples. **e** Difference in the RiskScore between the two groups, which were clustered by the expression of 17 genes

To further validate the stability as well as the reliability of the prognostic prediction model, the expression profile data of the abovementioned 17 genes were extracted from a total of 1068 samples, followed by substitution into the model. This was performed to calculate the RiskScore values for model validation, as previously described. The series of results are shown in Fig. 4. Taken together, the verification results based on the test set data suggested that the prognostic model established on the basis of the expression profiles of these 17 immune-related genes displayed excellent prediction accuracy and stability in identifying immune-related features.

**Fig. 5:**
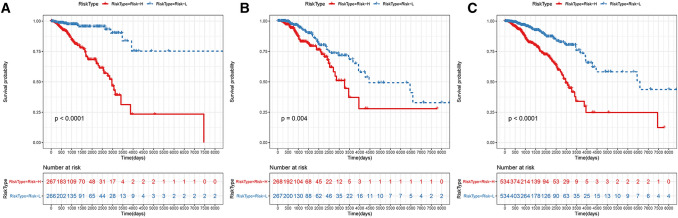
The KM survival curve of the 17-gene-based risk model in predicting the OS of the Risk-H and Risk-L groups in the training set (**a**), test set (**b**) and all samples (**c**)

Finally, the KM survival curves of the risk model, which were constructed based on the 17 genes in predicting the Risk-H and Risk-L groups for the training set, test set and all samples, are shown in Fig. 5. Figure 5a shows the KM survival curve of the training set (*p* < 0.0001), Fig. 5b shows the KM survival curve of the test set (*p* < 0.01), and Fig. 5c shows the KM survival curve of all the samples (*p* < 0.0001).

### Functional annotations of immune-related genes and signaling pathway enrichment specific to prognosis

**Fig. 6:**
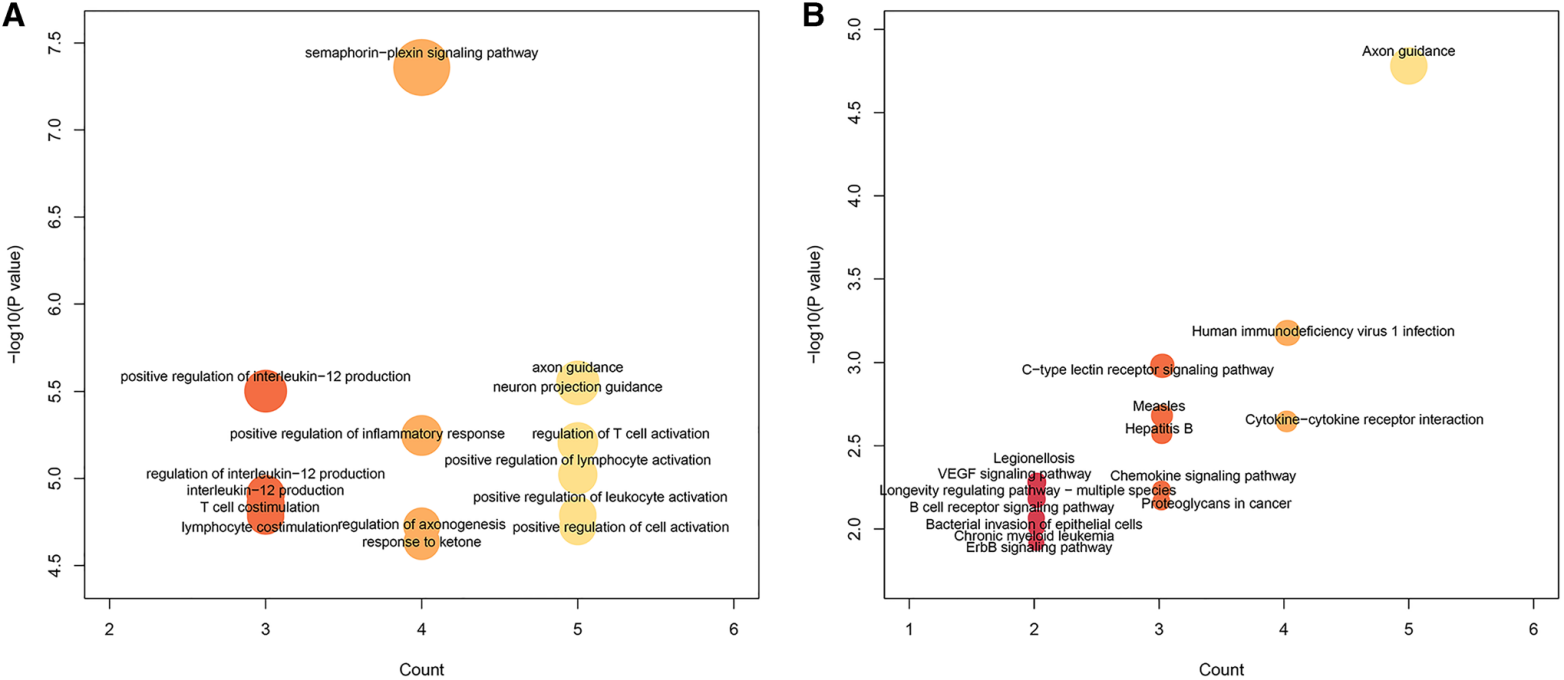
The GO (**a**) and KEGG pathway (**b**) enrichment analyses of the 17 specific immune-related genes

First, the gene families of the 17 obtained genes were annotated in accordance with the human gene classification in the HGNC database. As presented in Table 2, two genes were enriched into the Plexins family, and two genes were also significantly enriched in the Semaphorins family (*p* < 0.01). Moreover, the clusterProfiler package of R software was also used for the enrichment analyses of the 17 abovementioned immune-related genes specific to prognosis. The results of the GO enrichment are displayed in Fig. 6a, the results of the KEGG pathway enrichment analysis are presented in Fig. 6b, and data related to the GO and KEGG analyses are shown in Table S9 and Table S10, respectively. These results demonstrate that most of the abovementioned genes could be enriched in multiple immunity- and cancer-related biological processes and signaling pathways.

**Table 2:** 17-gene function annotation results

Gene family	Genes	*p* value	Padj
Plexins	PLXNB2/PLXND1	2.77E-05	0.000471545
Semaphorins	SEMA7A/SEMA3B	0.000115948	0.001971113
Type 2 receptor serine/threonine kinases	ACVR2A	0.004392947	0.074680102
Nuclear factors of activated T cells	NFATC2	0.004392947	0.074680102
Phosphatidylinositol 3-kinase subunits	PIK3CA	0.006582604	0.11190426
Toll-like receptors	TLR2	0.008039856	0.136677545
Apolipoprotein B mRNA-editing enzyme catalytic subunits	APOBEC3F	0.009495095	0.161416623
Heat shock 70-kDa proteins	HSPA2	0.013124409	0.223114961
Tumor necrosis factor superfamily	TNFSF4	0.013848769	0.235429069
Endogenous ligands	CCL22	0.155987949	1
Ankyrin repeat domain containing	NFKBIZ	0.164081906	1
Ring finger proteins	CBL	0.201688899	1
CD molecules	CCR7	0.253455841	1
Unknown	DAXX/TPT1	1	1

### Correlation of the RiskScore with the signaling pathways and clinical features of the samples

**Fig. 7:**
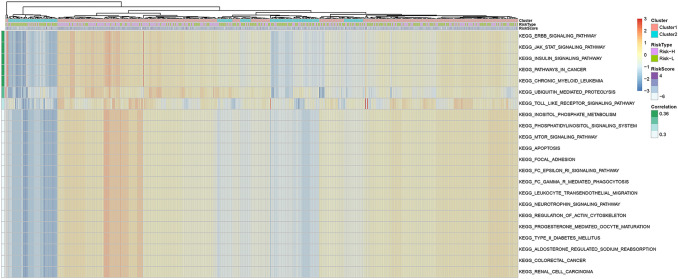
Correlation of the RiskScore with signaling pathways. The KEGG functional enrichment score of each sample was analyzed, and the correlation with the RiskScore was calculated based on the enrichment score of each pathway in each sample. The top 30 KEGG-related pathways are shown. The clustering analysis was performed according to the enrichment score in the training set

First, the KEGG functional enrichment scores of samples in the training set and test set and then those of all samples were analyzed using the ssGSEA function of the R software package GSVA. Moreover, the correlations with the RiskScore were also calculated according to the enrichment scores of all pathways in all samples. In all, 45 related KEGG pathways were obtained and are shown in Tables S11-S13. Among them, the top 50% of pathways were selected for the clustering analysis according to their enrichment scores, as shown in Fig. 7. The JAK/STAT signaling pathway, Insulin signaling pathway and Pathways in cancer had the best correlation with a correlation coefficient of approximately 0.36.

**Fig. 8:**
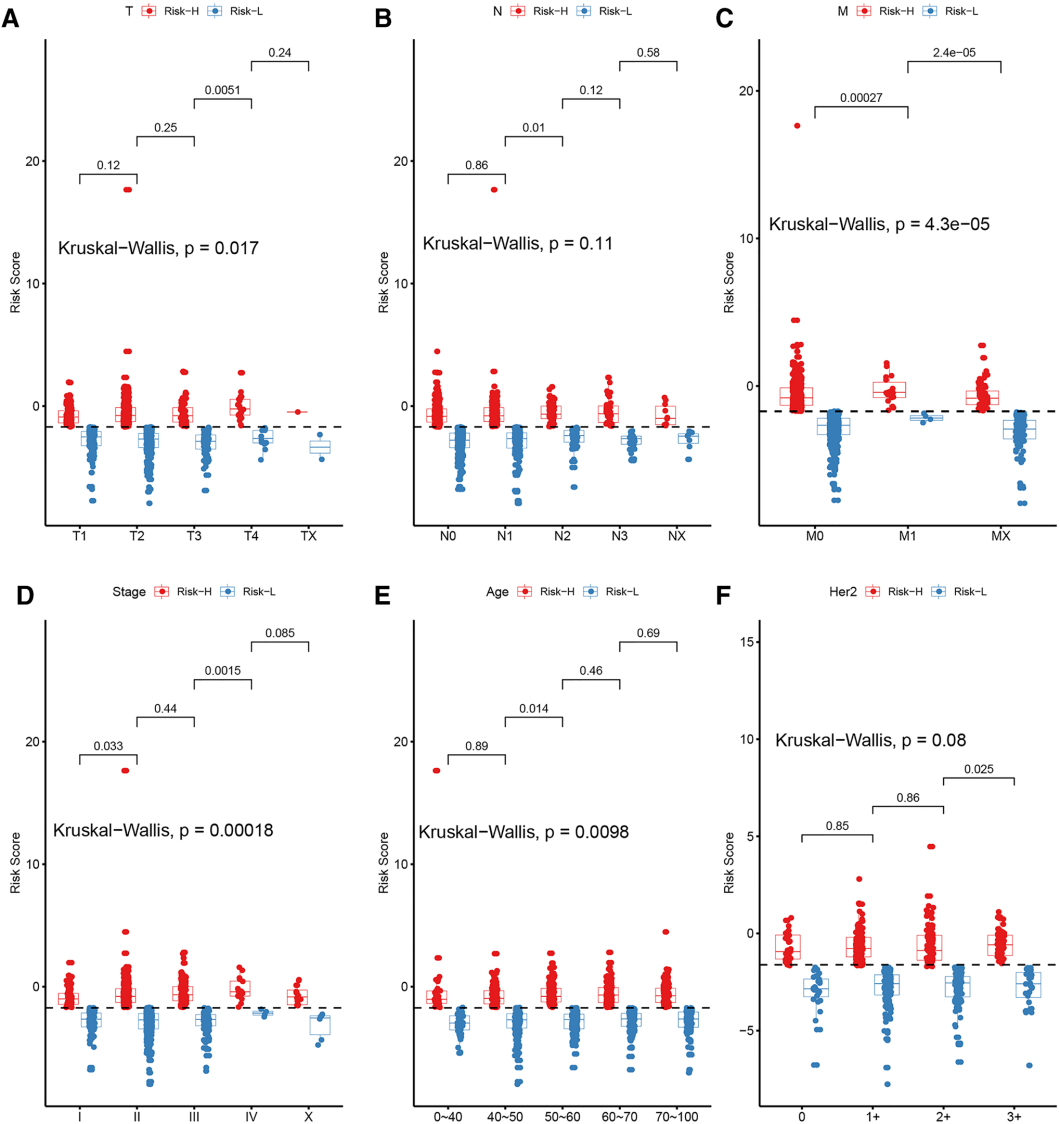
The relationship between different clinical factors and the RiskScore of BC patients. Comparison of the RiskScore for the different factors of T (**a**), N (**b**), M (**c**), stage (**d**), age (**e**) and Her2 expression status (**f**). The horizontal axis represents the different clinical factors, and the vertical axis represents the RiskScore

Thereafter, the correlations of various factors (including T, N, M, Stage, Age and HER2 expression) with the RiskScore were also analyzed, as shown in Fig. 8. Clearly, obvious associations were found between other features and the RiskScore (*p* < 0.05), which reveals that the RiskScore model was dependent on these clinical features.

**Fig. 9:**
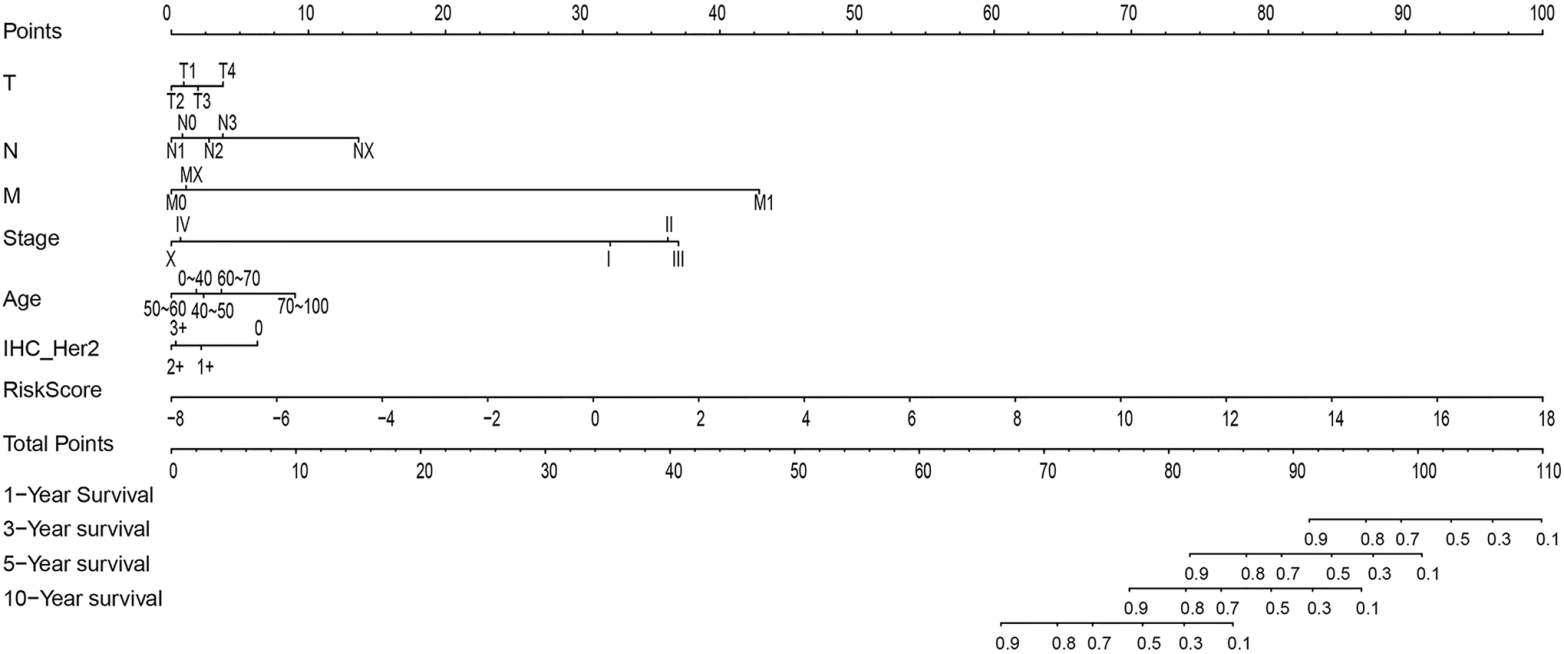
The nomogram model constructed by combining the clinical features (T, N, M, Stage, Age and Her2 expression) with the RiskScore of BC patients

On the contrary, the nomogram model was constructed using the RiskScore along with the clinical features. A nomogram is a method that can be used to intuitively and effectively demonstrate the results of a risk model, which can conveniently predict outcomes. In the nomogram, the straight-line length was used to examine the impacts of different variables (and their values) on the outcome. In this study, the nomogram model was established using the clinical features (including T, N, M, Stage, Age and HER2 expression) together with the RiskScore, as shown in Fig. 9. According to the model results, the RiskScore features remarkably affected the prediction of the survival rate, which indicates that the risk model based on the 17 genes could efficiently predict prognosis.

**Fig. 10:**
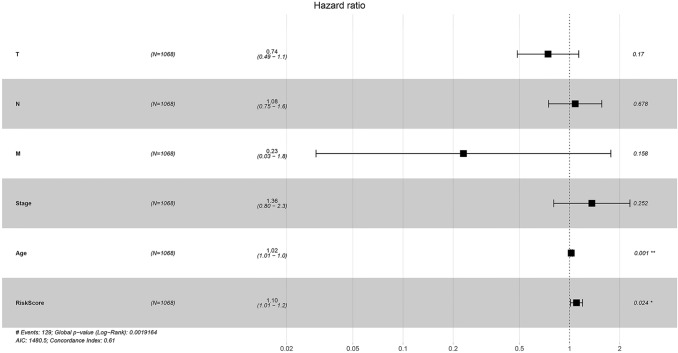
The forest plot constructed by combining the clinical features with the RiskScore of BC patients

Finally, the forest plot was established using both the RiskScore and the clinical features. Notably, the forest plot allows us to simply and intuitively illustrate the pooled statistical results of different research factors, which generally treats an ineffective line vertical to the *X*-axis (generally at the coordinate of X = 1 or 0) as the center, while several segments parallel to the *X*-axis represent the effect size and 95% confidence interval (CI) of each study. In this study, the forest plot was generated using the clinical features, such as T, N, Stage, Grade, Age, Alcohol consumption and Smoking status; the RiskScore was also calculated by the risk model, as shown in Fig. 10. The HR of the RiskScore was evidently increased compared with the HRs of other clinical features (*p* < 0.05). The multivariate Cox regression analyses of the various clinical features and the RiskScore are presented in Table S14.

## Conclusions

BC is a highly complex and heterogeneous malignancy that is associated with heterogeneous molecular profiles, clinical responses to therapeutics and prognoses [27]. Tumor heterogeneity is responsible for the various BC subtypes, which each have different prognoses and sensitivities to chemotherapy [28]. In addition, no consistent therapeutic benefits can be achieved among different patients from clinical medication, which can be ascribed to their potential toxicities and side effects. As a result, postoperative systemic adjuvant chemotherapy remains a source of controversy in clinical practice. Therefore, it is crucial to discover potential BC biomarkers that can predict patient prognosis and recurrence, as well as to administer early adjuvant chemotherapy to high-risk patients who may benefit [29].

BC has been recognized to be immunogenic, as it involves multiple putative tumor-associated antigens (TAAs), such as HER2 and Mucin 1 (MUC1) [30, 31]. Notably, over the last decade, these TAAs have been treated as targets for the development of new cancer vaccines and bispecific antibodies (bsAbs), among which, some have been translated into tumor-specific immune responses and have been verified to be clinically beneficial [32]. Immunocytes in BC tissue primarily consist of T-lymphocytes (70–80%), while the remaining components are derived from B lymphocytes, macrophages, natural killer cells and antigen-presenting cells (APCs) [33, 34]. Of these, T cells can be activated through recognition of the tumor antigens presented by APCs; typically, the intensity and quality of T cell activation signals are related to a variety of interactions between the receptor and ligand [35].

Substantial evidence has supported the concept that immunocytes in the tumor microenvironment can effectively enhance or suppress tumor growth, which can thereby serve as a prognostic indicator in BC patients. The interactions between the immune system and incipient cancer cells, which is also referred to as immunoediting, can be divided into 3 phases, namely, elimination, equilibrium, and escape [36]. Of these phases, the elimination process suggests that the innate and adaptive arms of the immune system will recognize the new antigens (derived from mutations or translocations) on the surface of incipient cancer cells, which is associated with MHC-I; alternatively, the distress signals can be expressed by the transformed cells with chromosomal changes (such as aneuploidy or hyperploidy). Finally, the immune system will eliminate these abnormal cells [37]. The equilibrium status will be reached when the immune system fails to eliminate the transformed cells but can stop them from further progression, and such a process has been deemed to be the dormancy phase during the development of primary cancer. This phase is mediated by the equilibrium between cells and cytokines (such as IL-12, IFN-γ, TNF-α, CD4 TH1, CD8 + T cells, NK cells and γδT cells) that promote elimination as well as those that promote the persistence of nascent tumors (including IL-23, IL-6, IL-10, TGF-β, NKT cells, CD4 Th2, Foxp3 + regulatory T [Treg] cells, and MDSCs) [38]. On the contrary, monocytes play a crucial role in this process, during which they may differentiate into proinflammatory M1 or anti-inflammatory M2 types as a result of the effects of the tumor microenvironment [39]. Immune escape of cancer cells may occur through various mechanisms. In HR-positive BC, the absence of strong tumor antigens and low MHC-I expression allow for tumor progression that is unnoticed by the immune system [40]. Estrogen exerts an immunosuppressive effect on the tumor microenvironment, which can boost tolerance to weak immunogenic cancers; moreover, estrogen receptor (ER) can be expressed on most immunocytes, including macrophages, T and B lymphocytes, and NK cells [41]. The immune response can be polarized to the Th2- rather than the Th1-effector immune response in the presence of estrogen [42, 43]. In HER2-positive cancer cells, MHC-I presentation is negatively correlated with HER2 expression [44]. Typically, triple-negative breast cancer (TNBC) exhibits a spectrum of MHC-I presentation and high antigen expression in the tumor, but immune escape in TNBC has been found to be predominantly related to the development of the immunosuppressive tumor microenvironment (including Tregs, MDSCs and PD-1/PD-L1) [45]. As a result, in the era of immunotherapy, it is particularly important to be familiar with the molecular events in the tumor-immune microenvironment to search for biomarkers related to survival prediction in patients with BC of any subtype.

In this study, 17 prognosis-specific immune-related genes were discovered through mining, statistics and sorting of big data such as that found in the TCGA and ImmPort databases; moreover, a prognostic prediction model was also constructed, and the RiskScore of the patients was calculated. Finally, prediction ability and verification were determined. Our findings suggest that the prognostic prediction model that was constructed based on the expression profiles of specific immune-related genes can further classify patients with a definite clinical stage into different subgroups based on the predicted survival results. Furthermore, the RiskScore is calculated according to the expression profiles of specific immune-related genes and should be used in combination with the clinical features of patients, which should more precisely predict BC patient survival. Taken together, this model may contribute to the identification of new BC markers in the clinic and can provide multiple targets for the precise medical treatment of BC. The model can also be used for the accurate classification of patients at the molecular subtype level. Finally, this model is promising in that it can guide clinicians in determining the prognosis, clinical diagnosis and appropriate therapy for BC patients with different immunophenotypes.

## Electronic supplementary material

Below is the link to the electronic supplementary material.

Supplementary file1 (TIF 162 KB)

Supplementary file2 (TIF 607 KB)

Supplementary file3 (TIF 1026 KB)

Supplementary file4 (TIF 530 KB)

Supplementary file5 (TIF 1113 KB)

Supplementary file6 (TIF 1012 KB)

Supplementary file7 (TXT 3420 KB)

Supplementary file8 (TXT 236 KB)

Supplementary file9 (TXT 19887 KB)

Supplementary file10 (TXT 9933 KB)

Supplementary file11 (TXT 9959 KB)

Supplementary file12 (XLSX 203 KB)

## Data Availability

The supplementary data used and generated during the current study are available from the corresponding authors on reasonable request.
