# Supplementary figures and images for: Immune-related gene data-based molecular subtyping related to the prognosis of breast cancer patients
Source: Breast Cancer. 2020 Nov 27;28(2):513–26. doi: 10.1007/s12282-020-01191-z (PMC7925489; doi:10.1007/s12282-020-01191-z)

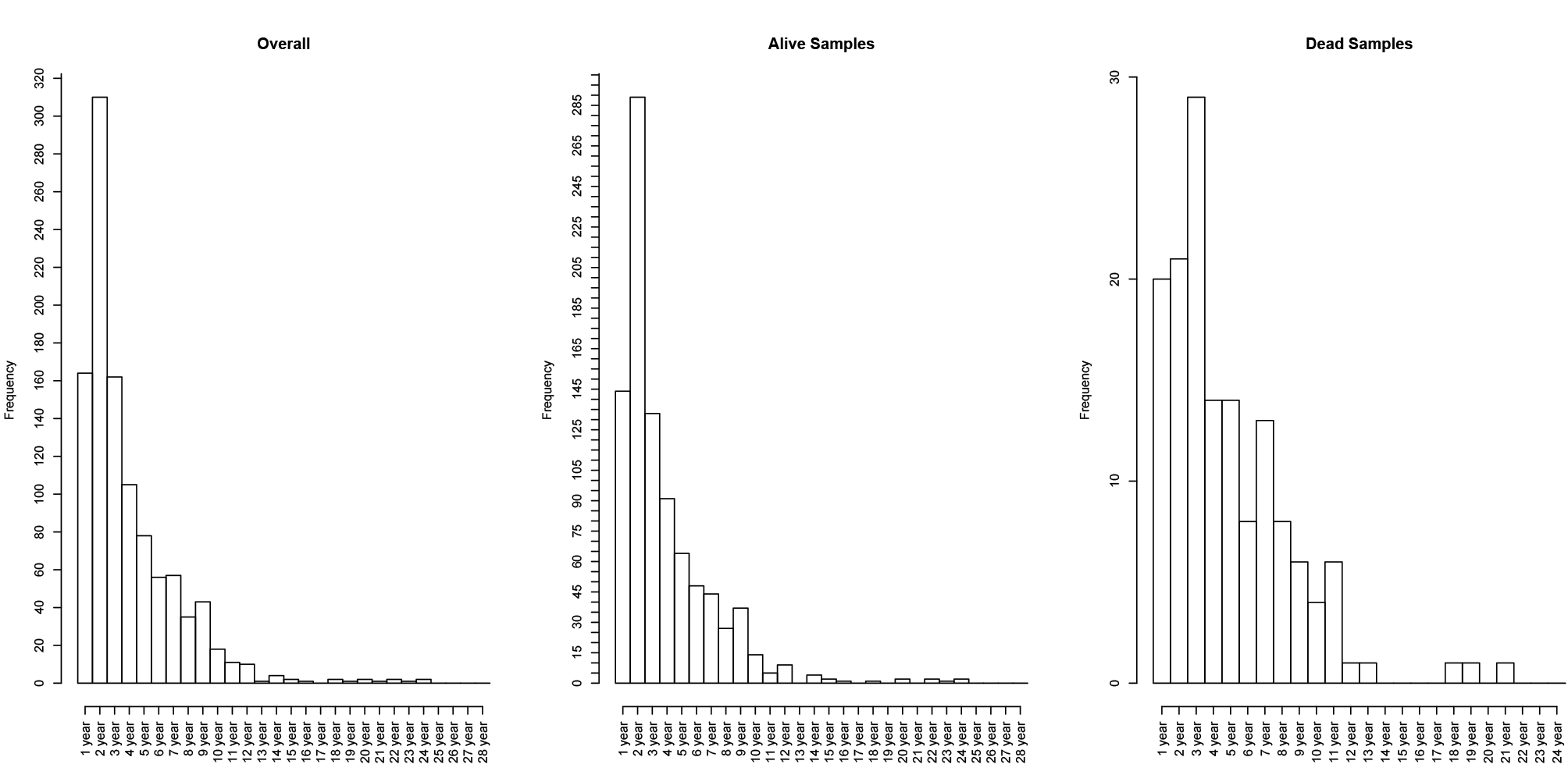

Supplement: Supplementary file 1 — Supplementary file1 (TIF 162 KB) [file 12282_2020_1191_MOESM1_ESM.tif]

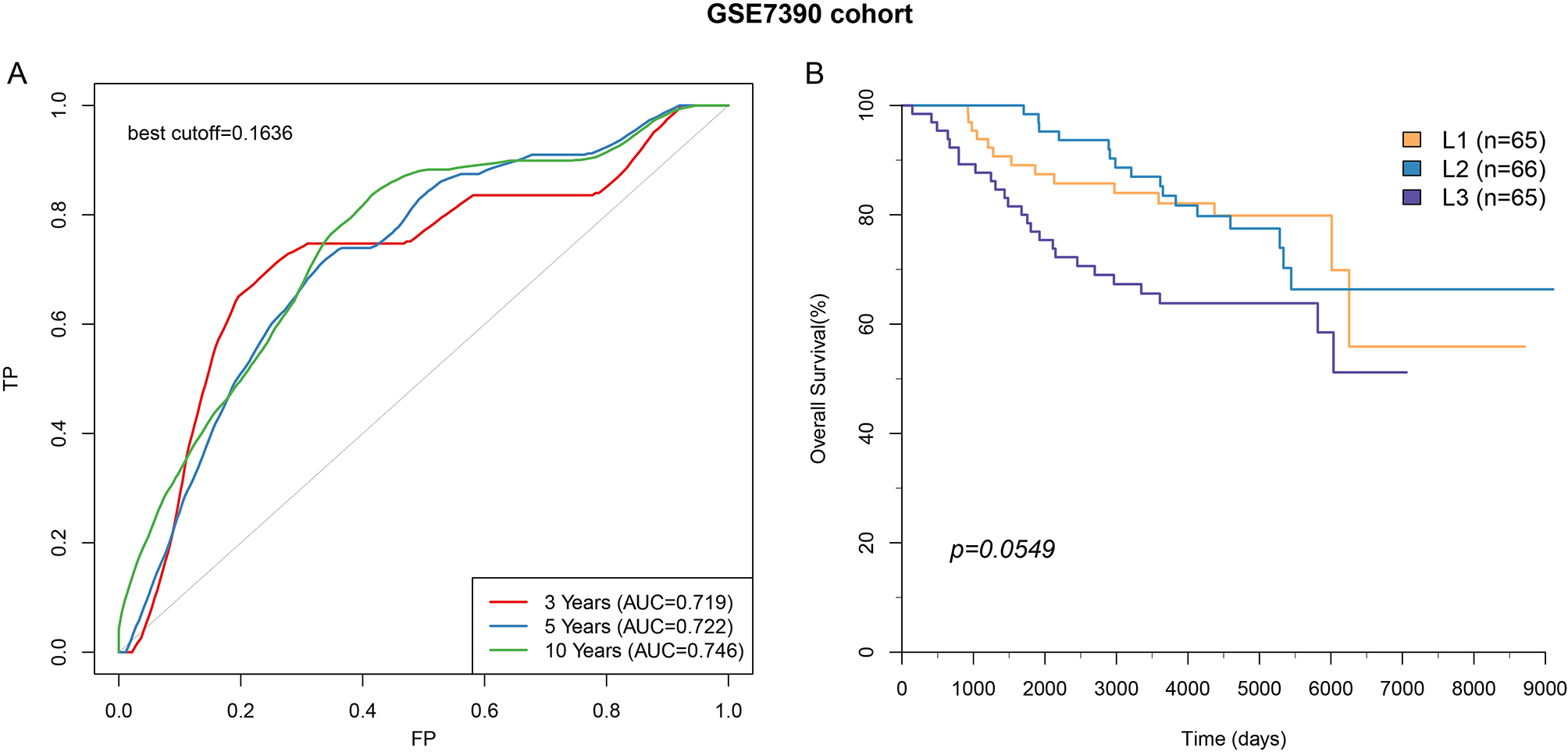

Supplement: Supplementary file 2 — Supplementary file2 (TIF 607 KB) [file 12282_2020_1191_MOESM2_ESM.tif]

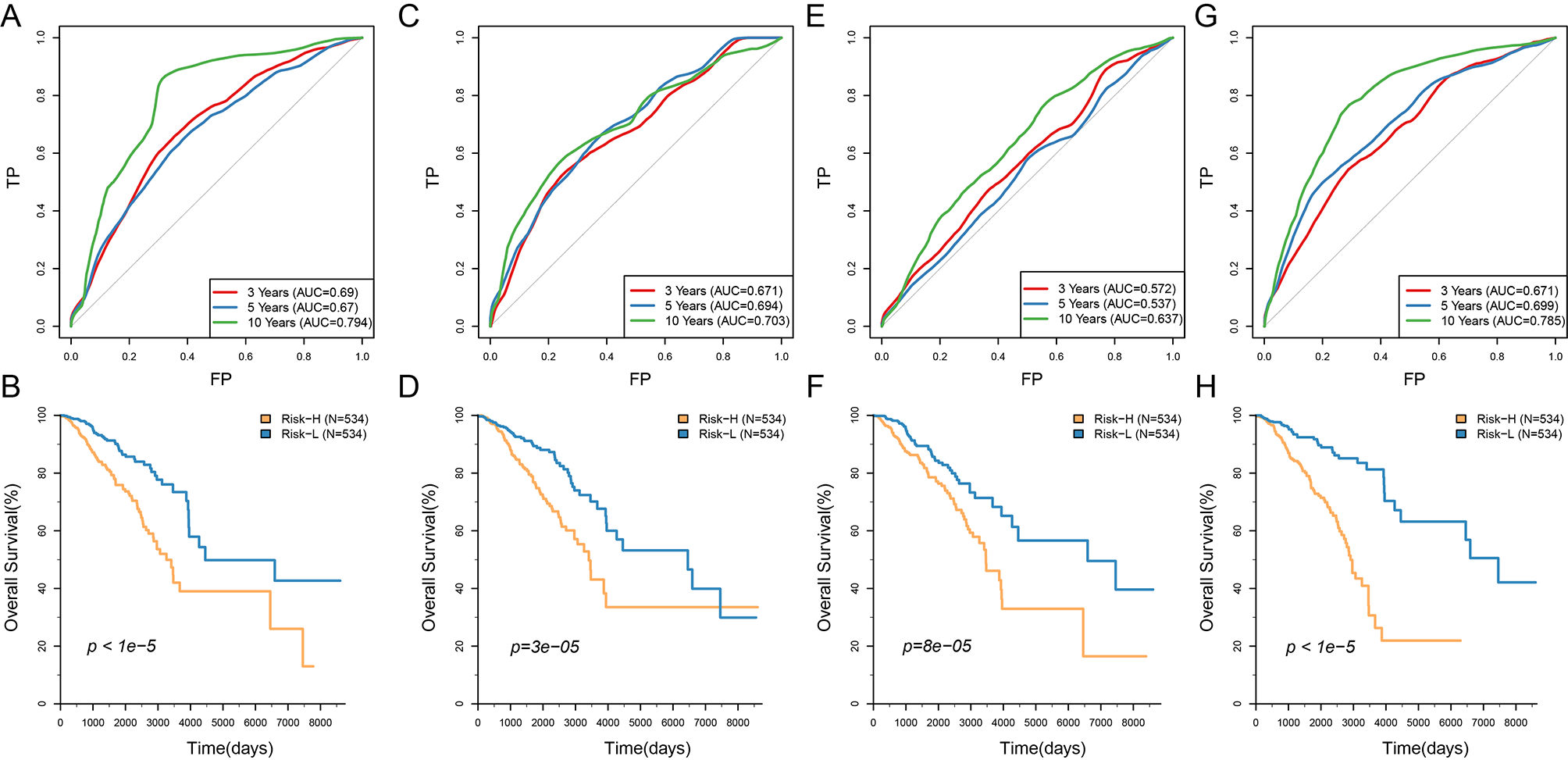

Supplement: Supplementary file 3 — Supplementary file3 (TIF 1026 KB) [file 12282_2020_1191_MOESM3_ESM.tif]

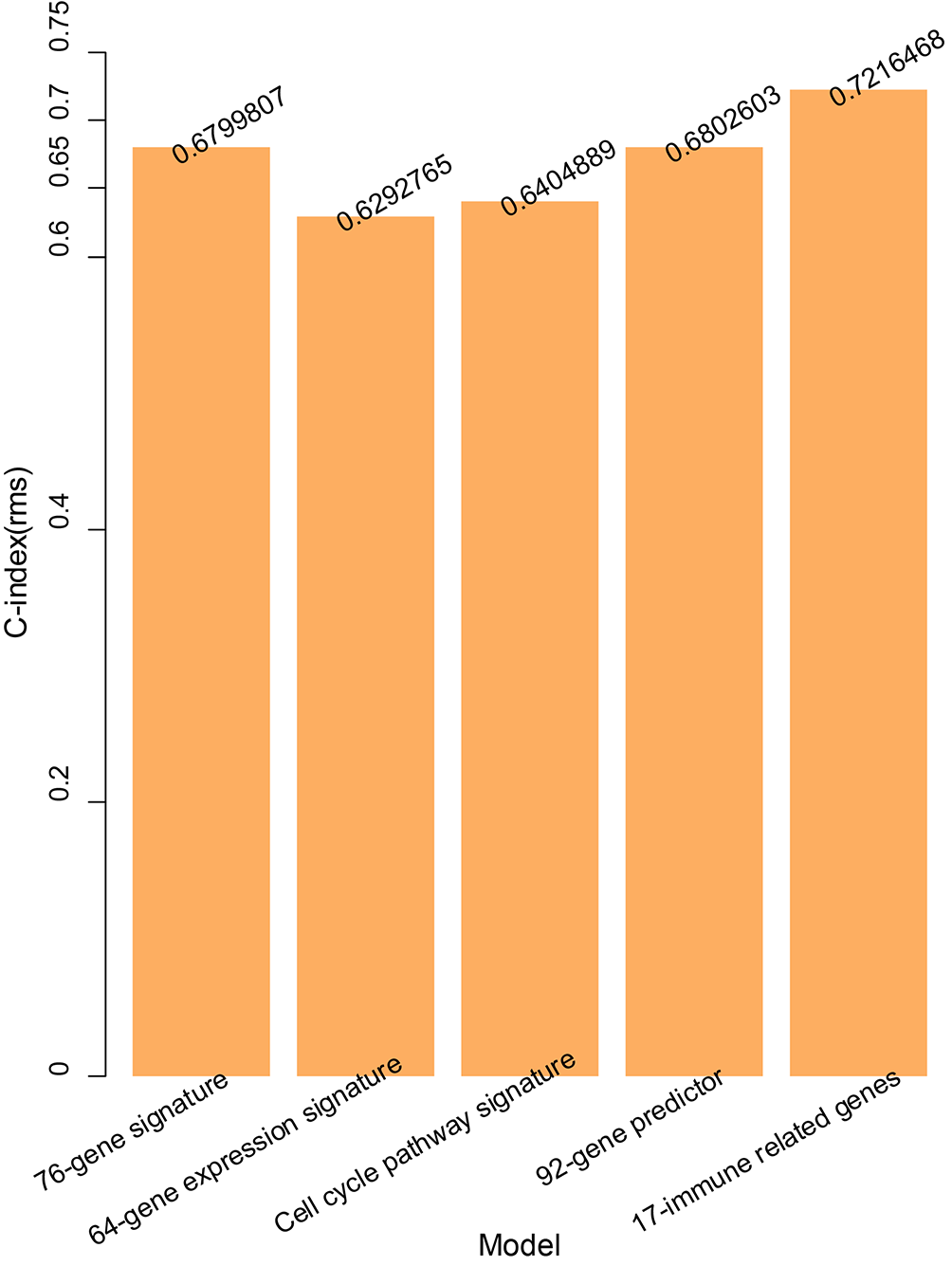

Supplement: Supplementary file 4 — Supplementary file4 (TIF 530 KB) [file 12282_2020_1191_MOESM4_ESM.tif]

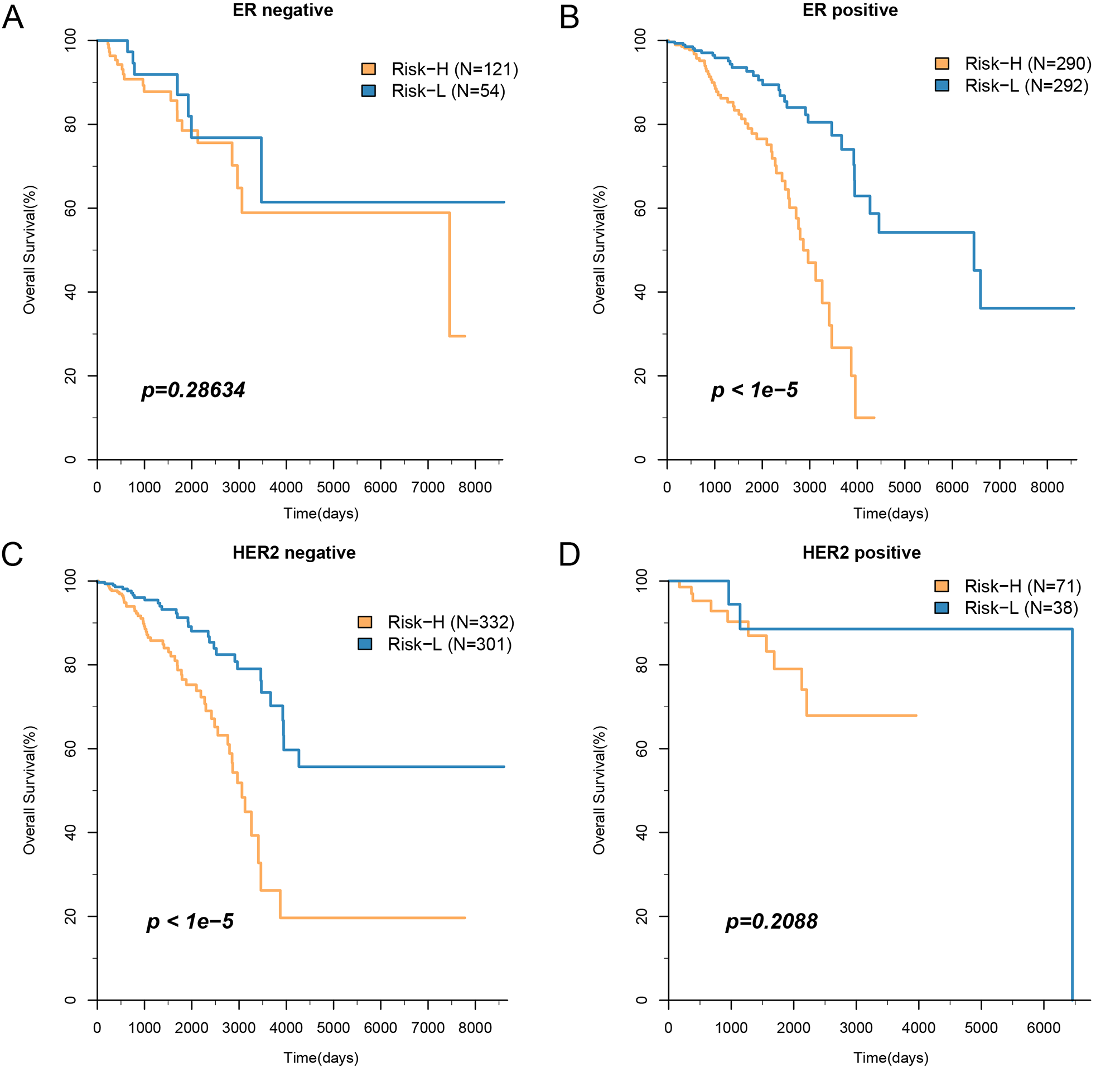

Supplement: Supplementary file 5 — Supplementary file5 (TIF 1113 KB) [file 12282_2020_1191_MOESM5_ESM.tif]

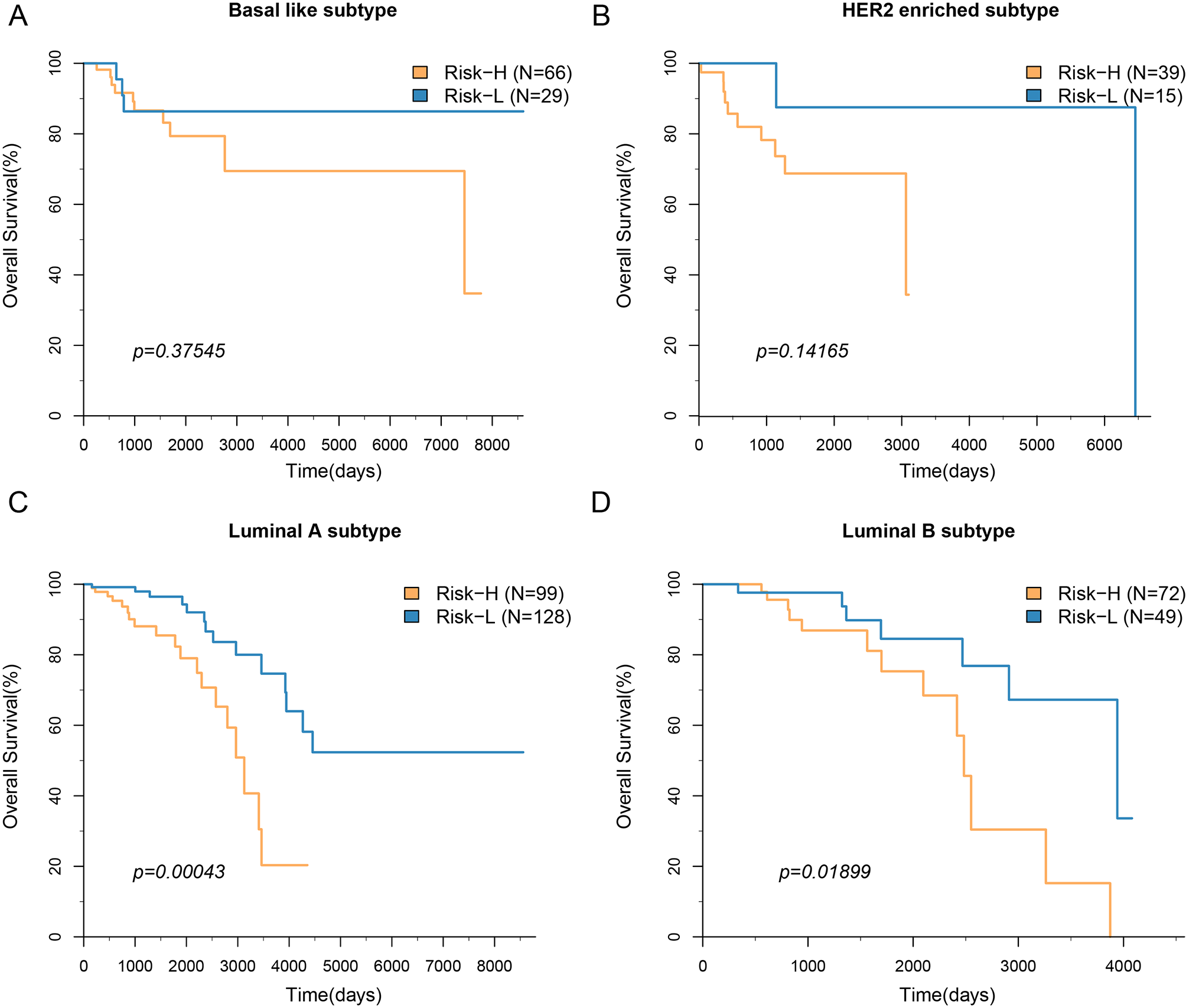

Supplement: Supplementary file 6 — Supplementary file6 (TIF 1012 KB) [file 12282_2020_1191_MOESM6_ESM.tif]
